# Structure of *N*-Terminal Sequence Asp-Ala-Glu-Phe-Arg-His-Asp-Ser of Aβ-Peptide with Phospholipase A_2_ from Venom of Andaman Cobra Sub-Species *Naja naja sagittifera* at 2.0 Å Resolution

**DOI:** 10.3390/ijms15034221

**Published:** 2014-03-10

**Authors:** Zeenat Mirza, Vikram Gopalakrishna Pillai, Wei-Zhu Zhong

**Affiliations:** 1 Proteomics and Structural Biology Unit, Fundamental and Applied Biology Group, King Fahd Medical Research Center, King Abdulaziz University, P.O. Box 80216, Jeddah 21589, Saudi Arabia; 2 Department of Biophysics, All India Institute of Medical Sciences, New Delhi 110029, India; E-Mail: vikgopal@gmail.com; 3 Children’s Hospital of Philadelphia, Philadelphia, PA 19104, USA; 4 Gordon Life Science Institute, Boston, MA 02478, USA; E-Mail: wzzhong@gordonlifescience.org

**Keywords:** cobra venom, phospholipase A_2_, co-crystallization, Alzheimer’s disease, neuroinflammation, DAEFRHDS

## Abstract

Alzheimer’s disease (AD) is one of the most significant social and health burdens of the present century. Plaques formed by extracellular deposits of amyloid β (Aβ) are the prime player of AD’s neuropathology. Studies have implicated the varied role of phospholipase A_2_ (PLA_2_) in brain where it contributes to neuronal growth and inflammatory response. Overall contour and chemical nature of the substrate-binding channel in the low molecular weight PLA_2_s are similar. This study involves the reductionist fragment-based approach to understand the structure adopted by *N*-terminal fragment of Alzheimer’s Aβ peptide in its complex with PLA_2_. In the current communication, we report the structure determined by X-ray crystallography of *N*-terminal sequence Asp-Ala-Glu-Phe-Arg-His-Asp-Ser (DAEFRHDS) of Aβ-peptide with a Group I PLA_2_ purified from venom of Andaman Cobra sub-species *Naja naja sagittifera* at 2.0 Å resolution (Protein Data Bank (PDB) Code: 3JQ5). This is probably the first attempt to structurally establish interaction between amyloid-β peptide fragment and hydrophobic substrate binding site of PLA_2_ involving H bond and van der Waals interactions. We speculate that higher affinity between Aβ and PLA_2_ has the therapeutic potential of decreasing the Aβ–Aβ interaction, thereby reducing the amyloid aggregation and plaque formation in AD.

## 1. Introduction

An estimated 36 million people globally are suffering from Alzheimer’s disease (AD), which causes irreversible neurodegeneration and usually strikes in the later years of life. According to the World Health Organization, this figure is anticipated to rise to 65.7 million by 2030 and may increase to 115.4 million by 2050 [1]. Dementia is the most frequent type of neurodegenerative disease and it is rarely detected before symptoms develop; no drugs presently exist for its therapy [2,3]. The characteristic disease landmarks include neurofibrillary tangles [4] and amyloid plaques, surrounded by reactive astrocytes, activated microglial cells causing neuroinflammatory responses and dystrophic neuritis. Although the neuroinflammation mechanism in AD brain is not apparent, there is ample data suggesting a role for specific forms of amyloid beta peptide (Aβ) in inducing release of pro-inflammatory cytokines by microglia and astrocytes. Hence, identifying the modulating mechanisms of neuroinflammatory responses and neuronal degeneration will unravel vital aspects to develop new therapeutic strategies [5,6]. Developing chemical interventions for AD is exigent and has proceeded in a virtual vacuum due to lack of tertiary structural information of amyloid-β peptide [7], which is cleaved via the β/γ-secretase pathway from the membrane-bound amyloid precursor protein (APP) [8]. β-secretase generates the *N*-terminus of Aβ by cleaving β-APP within the Glu-Val-Lys-Met-↓-Asp-Ala sequence or by cleaving the Swedish mutant β-APP_SW_ within the Glu-Val-Asn-Leu-↓-Asp-Ala sequence. In addition, cleavage has been reported to occur within the Aβ sequence Asp-Ser-Gly-Tyr^10^-Glu^11^-Val, generating Aβ_11–40/42_ [9]. Solubility may be modulated in a pH-dependent manner by the charged *N*-terminal sequence [10].

The phospholipase A_2_ (PLA_2_) is a lipolytic enzyme commonly expressed in several types of mammalian cells [11]. Two most notable forms of PLA_2_ are the secretory PLA_2_ (sPLA_2_) and the calcium-dependent cytosolic PLA_2_ (cPLA_2_). In healthy brain cells, equilibrium between arachidonic acid conversion into proinflammatory mediators and arachidonic acid reincorporation into the membrane is maintained by PLA_2_ regulation. Unregulated PLA_2_ activity causes production of an inconsistent amount of proinflammatory mediators, leading to oxidative stress and neuroinflammation as seen in neurological diseases such as AD, epilepsy, and multiple sclerosis. The most common and extensively studied PLA_2_s belong to group I and II. sPLA_2_-IIA mRNA is up-regulated in AD brains as compared to non-demented elderly brains, and a higher percentage of sPLA_2_-IIA-immunoreactive astrocytes associated with Aβ plaques have been reported in the AD hippocampus and inferior temporal gyrus [12]. Increased sPLA_2_ activity is observed in the cerebrospinal fluid of humans with AD and multiple sclerosis, and can perhaps be a marker of permeability increases of the blood–cerebrospinal fluid barrier [13]. Also, other types of sPLA_2_ bearing a similar structure—e.g., groups 1B, IIE, V and X—are present in distinct brain regions [14]. A feature identified for the design of tight PLA_2_ inhibitors is the presence of the OH group on the aromatic framework, which may be extended in the opposite direction with the hydrophobic moiety [15].

A series of recent studies have indicated that much useful information for drug development can be obtained in a timely manner by conducting various studies, either experimentally or theoretically. However, different targets would need different approaches. For instance, to reveal the molecular mechanism of Alzheimer’s disease [16–18] and find useful clues for developing drugs against Alzheimer’s disease [19,20], the structural bioinformatics tools [21] were adopted. On the other hand, as is well known, X-ray crystallography and high-resolusion NMR (see, e.g., [22–24]) are two very powerful tools for structure-based drug design. Although it is time-consuming and expensive to use these facilities, the results thus acquired are usually more reliable and dependable. Our primary goal is to determine the possibility of a direct interaction between Aβ peptide and PLA_2_ and the structure adopted by the peptide that may in the future pave the way for novel approaches for better understanding AD and its therapeutics.

## 2. Results

### 2.1. Quality of the Final Model

**Figure 1.**
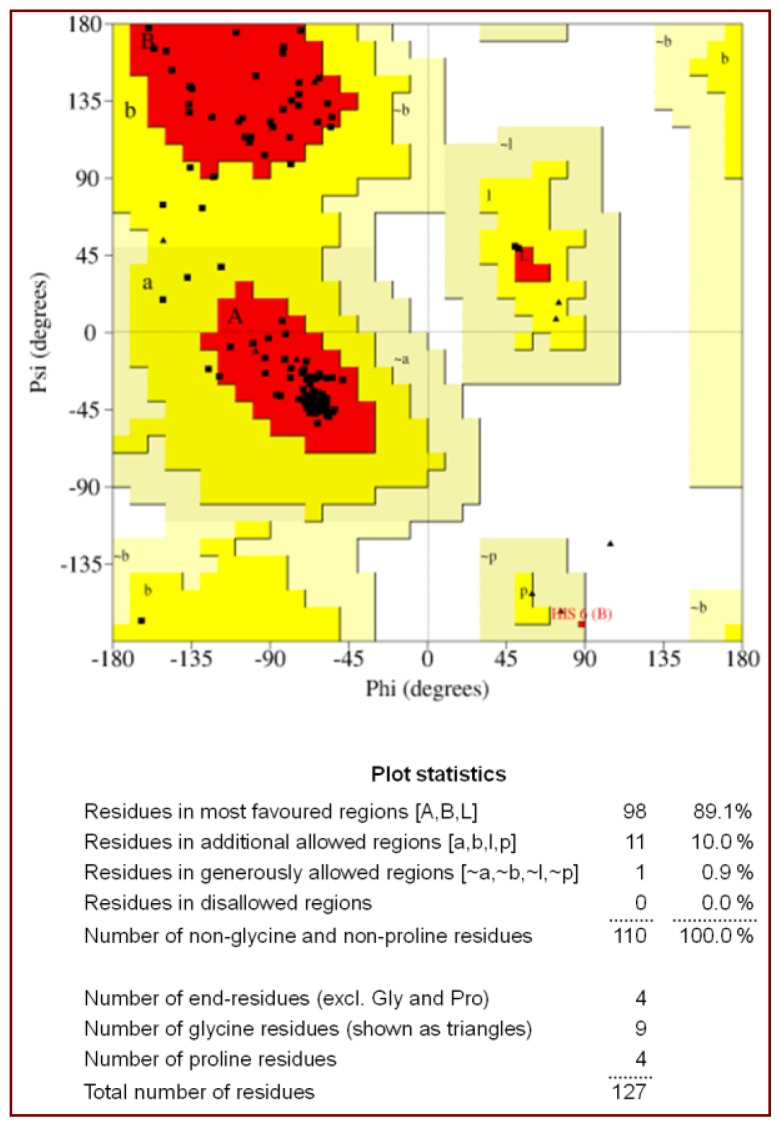
A Ramachandran plot of the main chain torsion angles (ϕ,ψ) for the final refined model. The plot was calculated with the program PROCHECK [25]; non-glycine residues are identified by squares.

The final model consists of 909 protein atoms, 68 atoms of peptide molecule, one calcium ion and 99 water molecules. The final |2F_o_ − F_c_| electron density map is continuous and well defined for both the backbone and the side chains of the protein. The final model has a good overall geometry with the r.m.s. deviations in bond lengths and angles are 0.009 Å and 1.1°, respectively. The Ramachandran plot calculated using PROCHECK [25], indicates that 89.1% of the residues are present in the most favourable regions, 10.0% were observed in the additionally allowed regions, while the remaining 0.9% residues were observed in the generously allowed regions of the Ramachandran plot [26] (Figure 1). The results of data collection and processing are given in Table 1 and the refinement statistics are given in Table 2.

### 2.2. Overall Structure

**Figure 2.**
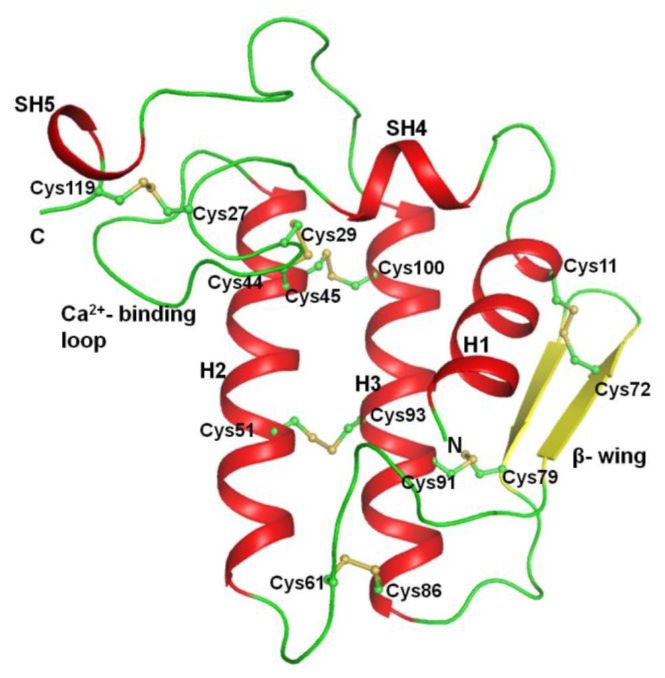
A ribbon diagram showing the overall structure of PLA_2_: helical segment is shown in red, β strands colored yellow and disulfide links shown in ball and stick, colored green and yellow. The three main helices are indicated as H1, H2 and H3, while two short helices are designated as SH4 and SH5. β wing, calcium-binding loop and disulfide linkages are also indicated.

**Figure 3.**
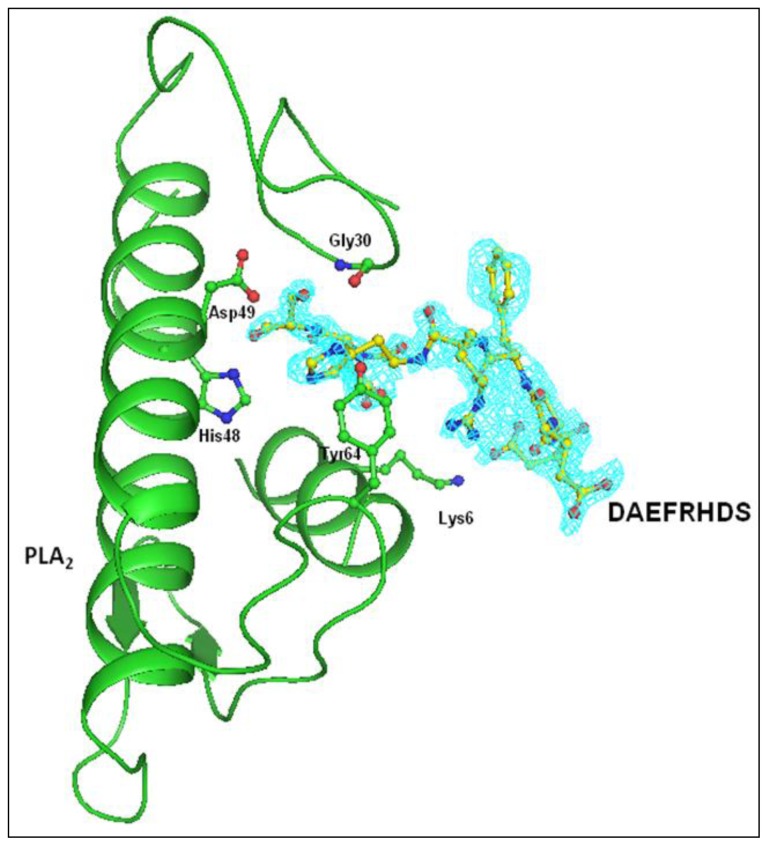
The |F_o_ − F_c_| electron density map contoured at 2.0 σ showing the electron density for the peptide Asp-Ala-Glu-Phe-Arg-His-Asp-Ser.

The general structure of PLA_2_ contains an *N*-terminal helix, H1 (residues: 2–12), a calcium-binding loop (residues: 25–35), a second α-helix, H2 (residues: 40–55), a short two-stranded antiparallel β-sheet (residues: 75–78 and 81–84), referred to as the β-wing and a third α-helix, H3 (residues: 90–108). There are two helical short turns involving residues 19–22 (SH4) and 113–115 (SH5) (Figure 2). The two antiparallel helices H2 and H3 form the core of the protein structure. The hydrophobic residues on the inner surface of the helix H1 are highly conserved and form one wall of the hydrophobic channel, which provides access to the catalytic site (Figure 3). Additional contributions to the hydrophobic channel include amino acid 19, which is located in the short turn following the helix H1, amino acid 30, 31 and 32 located within the calcium-binding loop and amino acid 69 located before the first strand of the β-wing. The structure is in accordance with previously reported structures [27].

**Figure 4.**
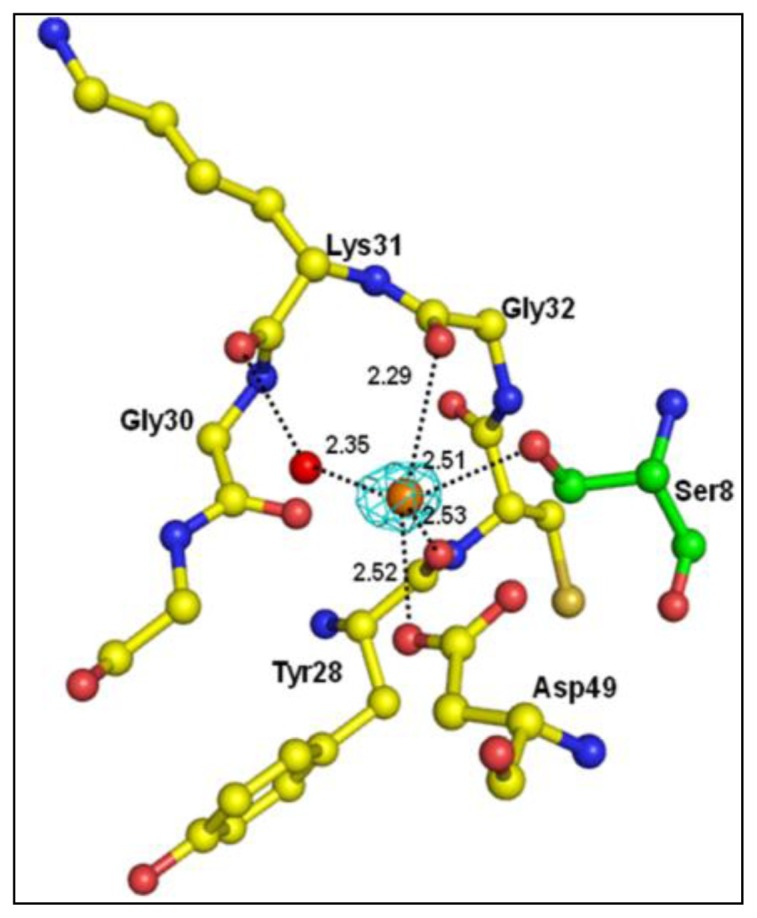
Difference |F_o_ − F_c_| electron density for the calcium ion drawn at 2σ. Calcium coordinated interactions are indicated by dotted lines. Ser8 of peptide is shown in green.

The overall folding of PLA_2_ observed in the complex with peptide is essentially similar to that of native PLA_2_ (1MF4) with an r.m.s. shift of 0.2 Å for the C^α^ positions. One milli molar CaCl_2_ was added in the protein drops that were used for crystallization and the structure revealed the presence of Ca^2+^ ion in the so-called calcium-binding loop. The Ca^2+^ ion is considered generally essential for catalytic activities of secretory PLA_2_s [28,29]. In the present structure, the Ca^2+^ ion stabilizes the conformation of the calcium-binding loop (Figure 4).

### 2.3. Structure of Peptide

**Figure 5.**
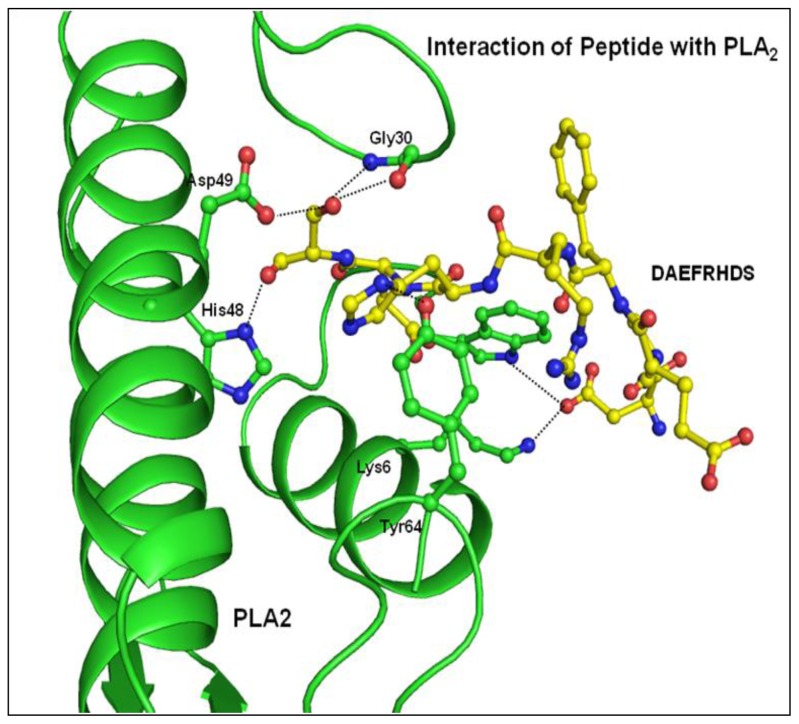
Interactions between PLA_2_ and the peptide Asp-Ala-Glu-Phe-Arg-His-Asp-Ser. The peptide residues are colored yellow. The critical interactions between peptide and protein are shown by the dotted line.

**Figure 6.**
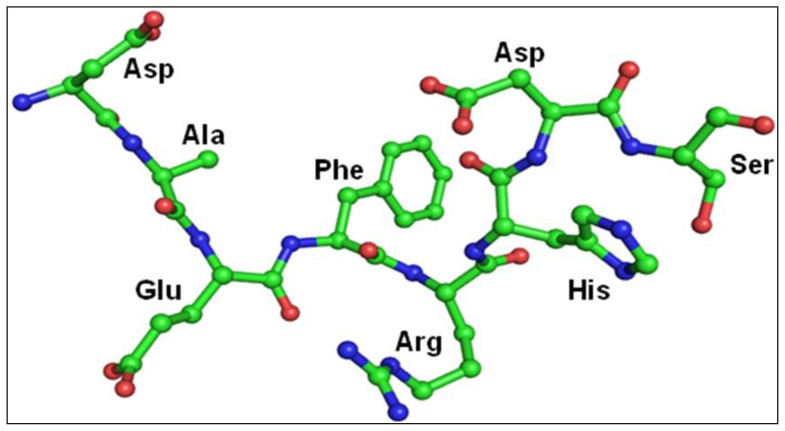
The structure of peptide Asp-Ala-Glu-Phe-Arg-His-Asp-Ser in complex with PLA_2_.

The structure of PLA_2_ in the complex remains unchanged from its native structure. All the eight residues of the peptide can be traced from their electron densities (Figure 3). The interaction of the peptide with the protein is depicted in Figure 5. Half of the peptide residue’s torsional angles are in the most favoured region of the Ramachandran plot, although none were observed in the disallowed region. The structure of the peptide is given in Figure 6.

## 3. Discussion

We have attempted the fragment assembly approach to elucidate the structure of Aβ. The fragment assembly and global optimization method has been established and extensively used in computational biology [30,31]. This work may be the first design of experiments following this approach. However, reductionist methods are common in protein crystallography. There are vast numbers of entries in PDB that are exclusively the domains, or even a small fragment of proteins. Most of the time, the intact protein is not amenable to crystallization, such as the beta-amyloid precursor protein. The co-crystallization method is another useful method of crystallography. Hundreds of Fab–Ag complexes are available to corroborate this fact. Co-crystallization of complete Aβ with mitochondrial alcohol dehydrogenase has been attempted [32]. The presence of Aβ in the crystal has been established by SDS-PAGE and *N*-terminal sequencing of the washed crystal in this study. However, no electron density corresponding to Aβ could be observed in the determined structure. This suggests the Aβ in this complex is flexible. The only instance where the Aβ molecule is seen in the crystal is the structure of the complex between Aβ and insulin-degrading enzyme (IDE). The Aβ is seen as a cleaved substrate. The complete molecule is not observed—only residues 1–3 and 17–22 are same [33].

There are many NMR studies describing the structures of partial and complete abeta molecule (3BAE, 1BA6, 1BA4, 2BEG). The results are generally combined with molecular modelling calculations. From all these studies, the following structural properties for the aggregating abeta is proposed—the central region Aβ_16–21_ and *C*-terminal region Aβ_33–40(42)_ are in β-strand conformation; Aβ_25–29_ is in loop conformation, and the rest of the molecule is in random conformation. This is also corroborated by X-ray fiber—diffraction of the fibrils while the attempts to crystallize or co-crystallize the Aβ_17–21_ and Aβ_35–40_/Aβ_37–42_ have been described and the peptides are observed in β-conformation. Apparently, the nature of binding sites of the protein influences the conformation of the Aβ peptide. The large space available in IDE accommodated the intact Aβ molecule. In 2OTK, [34], Aβ_17–36_ is seen in β-sheet conformation with residues 25–29 forming the loop. In their studies, Lustbader *et al*. could not view the Aβ molecule even though it was in the crystal. One conclusive aspect of crystal structures are that the peptides, Aβ_17–21_, Aβ_35–42_ and Aβ_17–36_ are in β conformation. This is in contrast to the solution studies that report all conformational possibilities. The same peptide has been observed in different conformations in different studies. Most of the studies report helical or coil conformation [35–38]. These results may be due to the variable solvent conditions used in these studies. Solvent conditions vary from completely polar to non-polar. The conformation of Aβ is highly dependent on the environmental conditions. Solvent polarity, temperature, pH and additives influence the solubility and aggregation behaviour of Aβ [39,40].

**Figure 7.**
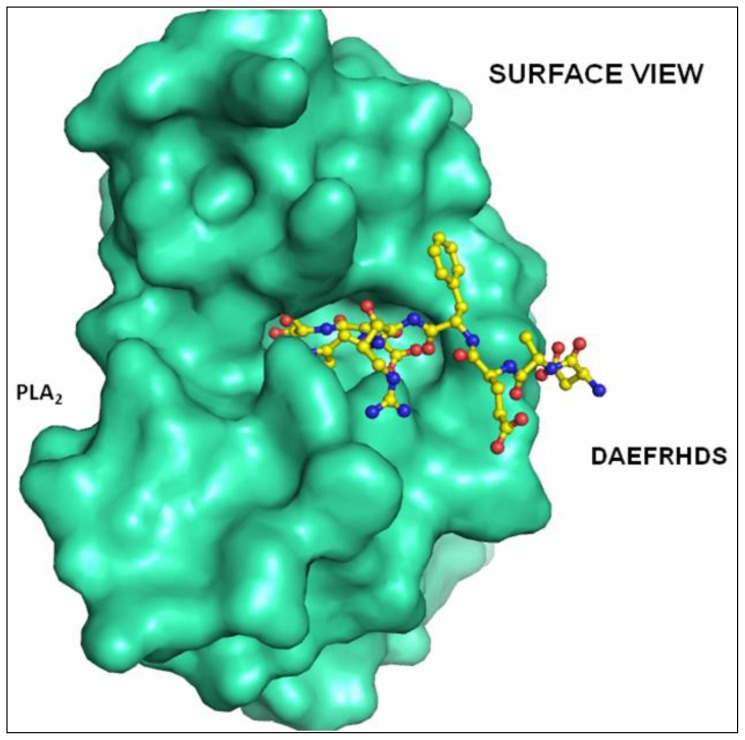
Surface diagram representation of the binding cavity and the hydrophobic channel with the peptide DAEFRHDS going inside the pocket.

An electron density was observed in the difference Fourier |F_o_ − F_c_| map in the complex structure (Figure 3), which allowed the interpretation of one molecule of the octa-peptide, as well as the detailed description of its interactions with PLA_2_. The peptide was positioned well in the hydrophobic channel (Figure 7) and was fitted well in the substrate binding site of enzyme. Peptide interacts with active site residues through a series of hydrogen bonds and hydrophobic interactions. The *N*-terminal part of the peptide lies towards the opening of the hydrophobic channel at the protein surface. The *C*-terminal serine residue is involved in hydrogen bonding with the active site residues. The rest of the peptide aligned in the hydrophobic channel makes a series of van der Waals contacts with protein atoms. The oxygen atom Oγ of Ser8 of peptide is hydrogen bonded with active site residue Asp49 Oδ1 and also with backbone atoms of Gly30 of the calcium-binding loop. The backbone oxygen atom of Ser8 is directly hydrogen bonded to His48 Nδ1. Thus the peptide interacts with the active site residues through direct hydrogen bonds (Table 3). The peptide also interacts with important residues of the hydrophobic channel like Asp1 residue of peptide interacts with Lys6 and Trp19 residue of protein and His6 residue of peptide interacts with Gly30 and Tyr64. Additionally the peptide is involved in van der Waals interaction with most of the residues lining the substrate-binding hydrophobic channel (Table 4).

In our studies, the binding site of the peptides on the protein is very hydrophobic. The binding cavity of PLA_2_ is lined with residues such as tryptophan, histidine, aspartic acid, and glycine. The non-polar surface extends from the molecule to the catalytic residues Aspartate and histidine at the other end. We expect that the non-polar nature of the binding site could have influenced the folding of the Aβ peptide fragment. This is possible given the fact that the folding of the Aβ molecule is mediated and stabilized by non-polar interactions. Moreover, the co-crystallization experiments were carried at 35% ethanol concentrations. Organic solvents (mostly alcohols have been studied), generally induce a random conformation in Aβ molecule as seen from the experiments. The peptide in our co-crystallization experiment strongly interacted with the non-polar binding-cavity residues of PLA_2_. The only interaction arginine displays in DAEFRHDS is non-polar (Table 3). Even though this peptide is polar it has more non-polar interactions than polar interactions (Tables 3 and 4). The observed conformation of the peptide in our result must have been dictated by the protein–ligand interactions. Though our aim of fragment assembly has not been achieved, the observations made by us are nevertheless interesting in their own right, exemplifying the strength of the interactions of the protein and ligand on one hand, and their effect on the conformation of the ligand on the other.

## 4. Experimental Section

### 4.1. Purification of Monomeric PLA_2_

The lyophilized samples of crude cobra venom of *Naja naja sagittifera* were obtained from Irula Snake Catchers Industrial Cooperative Society, Chennai, India. The crude venom was dissolved in 50 mM Tris-HCl, 100 mM NaCl, pH 7.0 at 100 mg/mL concentration and centrifuged at 12,000× *g* for 10 min to remove insoluble material. The collected supernatant was size fractionated on Sephadex G-100 column (100 × 2 cm) pre-equilibrated with 50 mM Tris-HCl, 100 mM NaCl, pH 7.0. The column was eluted with the same buffer at a flow rate of 6 mL/h. The peak corresponding to molecular weight of 14 kDa on SDS-PAGE and showing PLA_2_ activity was pooled for further purification. The pooled fractions were desalted and dialysed against 50 mM Tris-HCl, pH 7.0 and loaded on CM Sephadex C-50 column (Pharmacia, Uppsala, Sweden). The column was washed with the above buffer. The unbound fractions were pooled and dialysed against ammonium acetate buffer, pH 6.0. The diluted sample was loaded on a pre-equilibrated column with same buffer containing Affi-gel Cibacron blue F3GA. The column was washed with 50 mM ammonium acetate buffer pH 6.0 to remove unbound fractions. The column was eluted with 50 mM ammonium bicarbonate buffer pH 8.0. These fractions showed PLA_2_ activity and indicated a molecular weight of 14 kDa on SDS-PAGE. The samples were pooled, desalted by ultrafiltration using a 3 kDa cutoff membrane and lyophilized, and their purity was checked by matrix-assisted laser desorption-ionization–time of flight (MALDI-TOF) (Kratos, Shimadzu, Kyoto, Japan) and by activity measurements. On MALDI-TOF it showed a molecular weight of 13,401.99 Da. The protein samples were blotted on a polyvinyl difluoride (PVDF) membrane (Sigma-Aldrich, St. Louis, MO, USA) and were subjected to the *N*-terminal sequencing using an automated protein sequencer PPSQ-21A (Shimadzu, Japan). The *N*-terminal sequence of the first 15 residues was determined. It was found identical to the sequence of PLA_2_ whose structure was reported earlier [41].

### 4.2. Enzymatic Assay and Inhibition Studies

The purified enzyme was used for kinetic studies done using a PLA_2_ Assay Kit (Cayman Chemical Company, Ann Arbor, MI, USA). The enzymatic chromogenic assay utilized the conversion of arachidonoyl thio-phosphocholine into sulfahydryl molecule by PLA_2_. Arachidonoyl thio-PC is a synthetic substrate used to detect phospholipase activity [42]. Hydrolysis of the arachidonoyl thioester bound at the *sn*-2 position by PLA_2_ releases free thiol, colorimetrically detected by Ellman’s reagent [5,5′-dithiobis (2-nitrobenzoic acid) (DTNB)], which results in yellow colour along with the released sulfahydryl product. Stock concentration at 1.5 mM of PC-substrate and 0.1 mM of PLA_2_ were used for the assay. Ten μL of colouring agent (DTNB) was added in each assay reaction. Peptide inhibitors (GenScript Corporation, Piscataway, NJ, USA) were dissolved in dimethyl sulfoxide and only 5 μL added to the assay. Peptide concentrations of 0.10, 0.20, 0.30 and 0.40 mM were taken for studying PLA_2_ inhibition reactions. Bee venom PLA_2_ was taken as positive control. The assay included a 30 min pre-incubation of enzyme with peptide and a further incubation of 60 min at room temperature after the addition of 200 μL substrate solution. The absorbance was measured at 414 nm wavelength on a plate reader and measurements were repeated thrice. Two wells were designated as non-enzyme controls and their absorbance was subtracted from the absorbance measured in the sample wells. Significant decrease in enzyme activity was seen in the presence of an inhibitor.

### 4.3. Crystallization

The purified samples of PLA_2_ were dissolved in 10 mM sodium phosphate buffer pH 6.0 containing 1 mM CaCl_2_ to a final concentration of 2.5 mg/mL. Peptide was dissolved in the above buffer, containing 10% acetonitrile and added to the protein solution at 10-fold high molar concentration. The solution was incubated for 3 h, mixed well, centrifuged and kept for crystallization trials using hanging drop vapor diffusion method. The 10 μL drops of the above mixture were equilibrated against the same buffer containing 30% ethanol in the reservoir. The crystals grew to a size of 0.4 × 0.2 × 0.2 mm^3^ after two weeks.

### 4.4. Data Collection and Data Processing

The crystals of the complex formed between PLA_2_ and the *N*-terminus fragment DAEFRHDS were used for data collection at low temperature. A single crystal was mounted in a nylon loop and flash-frozen in a stream of nitrogen gas at 100 K. The data were collected on a 345 mm diameter MAR research scanner with 1.54 Å radiation generated by a Rigaku RU-300 rotating anode X-ray Generator filled with Osmic mirrors (Rigaku USA, Woodlands, TX, USA). The data were processed with DENZO and SCALEPACK from HKL package [43]. The final data set was complete to 87.7% up to 2.0 Å resolution. The crystals belong to the tetragonal space group P4_1_ with unit cell dimensions a = b = 42.7 Å, c = 65.8 Å. The presence of one molecule per asymmetric unit gave a crystal volume per protein mass (*V*_m_) of 2.3 Å^3^Da^−1^ corresponding to a solvent content of 46.7%. The final data show an overall completeness of 98.7% with a R_sym_ of 7.0% to 2.0 Å resolution (Tables 1 and 2).

### 4.5. Structure Determination and Refinement

The crystal structure was determined with molecular replacement method using auto-AMoRe [44] from the CCP4 software suit (Collaborative Computational Project, Number 4, 1994). The coordinates of a native PLA_2_ structure (PDB code: 1MF4) were used as a search model. The rotation and translation functions calculated with data in the resolution range, 12.0–3.5 Å yielded a unique solution with the first peak being very distinct. The stacking arrangement of the molecules in the unit cell for this solution yielded no unfavourable intermolecular contacts in space group P4_1_, thus confirming it as the correct space group. The coordinates were transformed using AMoRe and were then subjected to 20 cycles of rigid-body refinement with REFMAC5 [45]. This reduced the R_cryst_ and R_free_ factors to 18.1% and 22.0%, respectively. Of the reflections, 2% were used for the calculation of R_free_, and were not included in the refinement. The manual model building of the protein using Fourier |2F_o_ − F_c_| and difference Fourier |F_o_ − F_c_| maps was carried out with the Graphics Program “O” [46] on a Silicon Graphics O_2_ Workstation (Figure 2). A continuous non-protein electron density at 2.0 σ cut off was observed in the proximity of the active site that extended in a direction parallel to helix H_2_. The ligand was only included because it was well defined by unbiased difference Fourier (*i.e.*, before inclusion of any ligand) |F_o_ − F_c_| map. The coordinates of the peptide structure were fitted into the characteristic electron density (Figure 3). Water molecules were then added using ARP/WARP [45]. The presence of calcium ions was detected from the difference Fourier |F_o_ − F_c_| maps (Figure 4). Further refinement was carried out after adding the coordinates of the peptide molecule, one calcium ion and 99 water molecules. The final R_cryst_ and R_free_ factors for the complete data in the resolution range of 20.0–2.03 Å were 0.188 and 0.202, respectively (Table 2). A portion of the electron density indicating the quality of the structure at 2.03 Å resolution is shown in Figure 3. The atomic coordinates of this structure have been deposited to protein data bank (PDB) with an accession code of 3JQ5.

## 5. Conclusions

This is likely the first attempt to structurally establish the interaction between the amyloid-β peptide fragment and PLA_2_ peptide to the hydrophobic substrate binding site of PLA_2_ involving at least nine H bond and several van der Waals interactions. Higher affinity between Aβ and PLA_2_ decreases the Aβ–Aβ interaction probability, thereby reducing the aggregation and subsequent plaque formation. In conclusion, this study is a step towards understanding the mechanism behind the Aβ and PLA_2_ interaction that may facilitate the development of novel therapeutic strategies to inhibit inflammatory responses to retard many diseases.

## Figures and Tables

**Table 1.** Data collection statistics.

Space group	P4_1_
System	Tetragonal

Unit-cell parameters (Ǻ)	
a = b	42.7
c	65.8
*V*m (Ǻ ^3^/Da)	2.3
Solvent Content (%)	46.7
Resolution range (Ǻ)	20.0–2.0
No. of observed reflections	33,510
No. of unique reflections	7735
Overall completeness (%)	98.7
Completeness in the highest shell (2.06–2.03 Ǻ) (%)	87.7
Overall R_sym_ (%)	7.0
R_sym_ in the highest shell (2.06–2.03 Ǻ) (%)	18.8
Overall I/σ(I)	11.1
I/σ(I) in the highest shell (2.06–2.03 Ǻ)	2.3

**Table 2.** Refinement statistics.

PDB code	3JQ5
Resolution range (Ǻ)	20.0–2.0
Number of reflections	7735
R_Cryst_ (for all data) (%)	18.1
R_Free_ (5% data) (%)	22.0
Number of protein atoms	909
Number of peptide atoms	68
Number of Water Molecules	99
Number of calcium atoms	1

**R.m.s. deviations**	

Bond length (Ǻ ^2^)	0.009
Bond angles (°)	1.1
Dihedral angles (°)	14.4
Overall *G* factor	0.05

**Mean B factor (**Ǻ**^2^)**	

Main chain atoms	22.0
Side chains and water molecules	27.3
Overall	24.8

**Ramachandran plot statistics**	

Residues in the most allowed region (%)	89.1
Residues in the additionally allowed region (%)	10.0
Residues in the generously allowed region (%)	0.9

**Table 3.** Hydrogen bonds between PLA_2_ and peptide DAEFRHDS.

Atoms of peptide	Protein atoms	Distance (Å)
Asp1 Oδ2	Lys6 Nζ	3.35
	Trp19 Nɛ1	2.64
His6 Nδ1	Gly30 O	3.43
	Tyr64 OH	2.78
Ser8 Oγ	Gly30 N	3.22
	Gly30 O	2.62
	Tyr28 O	3.35
	Asp49 Oδ1	2.95
Ser8 O	His48 Nδ1	2.80

**Table 4.** Van der Waal interactions between PLA_2_ and peptide DAEFRHDS.

Atoms of peptide	Protein atoms	Distance (Å)
Asp1 Cγ	Trp19 Cɛ2	3.79
	Trp19 Cζ2	3.48
Ala2 Cβ	Trp19 CH2	3.73
	Trp19 Cζ2	3.97
Phe4 Cδ1	Ala23 Cβ	3.91
Arg5 Cζ	Leu2 Cδ2	3.53
His6 Cα	Leu2 Cδ2	3.69
His6 Cβ	Gly30 Cα	3.63
	Gly30 C	3.68
His6 Cɛ1	Tyr64 Cζ	3.75
Asp7 Cα	Ala23 Cα	3.75
Asp7 Cβ	Ile9 Cδ1	3.78
	Phe5 Cɛ2	3.97
Asp7 C	Phe5 Cɛ2	3.92
Ser8 Cβ	Tyr28 C	3.90
	Cys29 Cα	3.51
	Cys29 C	3.64
	Gly30 Cα	3.76
Ser8 C	Phe101 Cζ	3.92
	His48 Cγ	3.98
	Cys45 Cβ	3.81
